# Identification and Quantification of Activities Common to Intensive Care Patients; Development and Validation of a Dual-Accelerometer-Based Algorithm

**DOI:** 10.3390/s23031720

**Published:** 2023-02-03

**Authors:** Yvonne Dikkema, Noor Mouton, Koen Gerrits, Tim Valk, Mariëlle van der Steen-Diepenrink, Hans Eshuis, Han Houdijk, Cees van der Schans, Anuschka Niemeijer, Marianne Nieuwenhuis

**Affiliations:** 1 Association of Dutch Burn Centers, Burn Center Martini Hospital Groningen, 9728 NT Groningen, The Netherlands; 2 Research Group Healthy Ageing, Allied Healthcare and Nursing, Hanze University of Applied Sciences Groningen, 9714 CA Groningen, The Netherlands; 3 Department of Human Movement Sciences, University Medical Center Groningen, University of Groningen, 9700 GZ Groningen, The Netherlands; 4 Department of Neuroscience, University Medical Center Groningen, University of Groningen, 9700 GZ Groningen, The Netherlands; 5 Department of Intensive Care, Martini Hospital, 9728 NT Groningen, The Netherlands; 6 Burn Center, Martini Hospital, 9728 NT Groningen, The Netherlands; 7 Department of Rehabilitation Medicine, University Medical Center Groningen, University of Groningen, 9700 GZ Groningen, The Netherlands; 8 Department of Health Psychology, University Medical Center Groningen, University of Groningen, 9700 GZ Groningen, The Netherlands

**Keywords:** accelerometer, ICU, mobilization, rehabilitation, validity, algorithm, wearable technology, activity

## Abstract

The aim of this study was to develop and validate an algorithm that can identify the type, frequency, and duration of activities common to intensive care (IC) patients. Ten healthy participants wore two accelerometers on their chest and leg while performing 14 activities clustered into four protocols (i.e., natural, strict, healthcare provider, and bed cycling). A video served as the reference standard, with two raters classifying the type and duration of all activities. This classification was reliable as intraclass correlations were all above 0.76 except for walking in the healthcare provider protocol, (0.29). The data of four participants were used to develop and optimize the algorithm by adjusting body-segment angles and rest-activity-threshold values based on percentage agreement (%Agr) with the reference. The validity of the algorithm was subsequently assessed using the data from the remaining six participants. %Agr of the algorithm versus the reference standard regarding lying, sitting activities, and transitions was 95%, 74%, and 80%, respectively, for all protocols except transitions with the help of a healthcare provider, which was 14–18%. For bed cycling, %Agr was 57–76%. This study demonstrated that the developed algorithm is suitable for identifying and quantifying activities common for intensive care patients. Knowledge on the (in)activity of these patients and their impact will optimize mobilization.

## 1. Introduction

For inpatient individuals who receive intensive care (IC), including those with extensive burns, physical functioning is severely impaired. This can lead to many secondary problems such as ICU-acquired weakness and other physical, cognitive, or mental disorders. Being physically active as a form of rehabilitation, even in the early stages of recovery, is important for optimizing physical functioning and increasing autonomy [1]. Therefore, adequate physical activity in the IC setting is important for improving physical functioning [2,3]. However, what constitutes “adequate” physical activity has yet to be determined. To gain an understanding of what levels of physical activity are adequate for rehabilitation, knowledge about the actual physical activity levels in terms of frequency, duration, and type of activity, i.e., external load, during a hospital stay is essential [4,5]. Unfortunately, such knowledge is mostly lacking.

For intensive care patients, several methods exist for monitoring physical activities, including mobility scales, behavioral mapping, and the use of wearable activity monitors [6]. Mobility scales are easy to score and require only a minimal amount of equipment. However, they do not provide insight into the type, duration, and frequency of actual physical activity nor the continuity of it [7]. Behavioral mapping with video observations, for example, provides this information in a valid and reliable manner. However, it is very labor intensive and therefore not practical to conduct for longer periods of time [8]. Wearable activity monitors, such as accelerometers, enable continuous objective measurements for extended periods of time in hospitalized patients, including those in intensive care [5,9]. In addition, they are small, relatively inexpensive, and well tolerated. However, the disadvantage of using accelerometers is that, when applied singularly, they do not allow for the differentiation of physical activities common to hospitalized intensive care patients, such as lying and sitting [10,11]. To overcome this issue, dual accelerometry using two accelerometers at the same time combined with an algorithm based on the raw three-axial data of both of these can be used [5,12,13].

To date, several algorithms for use in clinical settings based on dual-accelerometry have been developed. They can identify older adults’ activities, such as sitting, standing, and lying [14] as well as slow walking by elderly patients in a clinical setting [15]. Additionally, Rauen et al. [16] have developed an algorithm that can identify activities such as supine lying, side lying, or sitting in severely affected neurological patients. Although these algorithms identify many activities, they do not include other activities that are common for IC patients such as nurse-initiated activities, i.e., transfers with the assistance of a healthcare provider, semi-sitting in a bed, and transitions between those activities or bed cycling. Furthermore, although the dual-accelerometer-based method combined with an algorithm has the potential to identify many activities, there are still some considerations regarding the fundamentals of the algorithm and wear locations. Concerning the algorithm, its development can be based on multiple variables, such as body movement, different body angles, or gravitational segments. As a result, each developed algorithm is very specific to certain physical activities and patient population [17], and a validated algorithm related for a specific purpose is of immense importance. With regard to the wear location, it was shown that this may influence the ability to identify different activities [18]; accelerometers placed on the leg are less able to ascertain activities being performed by the body [16] and may be better for detecting walking [10]. The presence of, for example, burns or lines on different locations of the body could influence where the accelerometers can be placed; however, it is not clear whether various locations require different algorithms.

Thus far, there is no algorithm that is validated for identifying the activities common for IC patients. Given the need to understand what constitutes adequate physical activity for IC patients, we aimed at developing and validating a dual-accelerometer-based algorithm to identify and quantify activities common for IC patients.

## 2. Methods

### 2.1. Design

A cross-sectional observational study was conducted in January and February 2021 in a Dutch hospital with a dedicated burn center (Martini Hospital, Groningen, The Netherlands). The study was approved by the ethics committee of Martini Hospital (no. 2020-141).

### 2.2. Participants

Eligible participants were those who could understand Dutch, were older than 18 years, and had no physical limitations that would affect the activities required in this study. They were recruited via e-mail, telephone, or personal contact. Only employees of the Martini Hospital could participate, due to COVID-19 restrictions.

To provide insight into the heterogeneity of the sample, data on gender and age (years) were collected, and participants’ height (centimeters) and weight (kilograms) were measured. Before the study began, they were afforded the opportunity to ask questions. All participants gave informed consent prior to the initiation of the study. Based on the literature on the validation of algorithms for accelerometer data, we aimed for 10 participants [13,18,19,20].

Data from the ten participants were randomly allocated to be used for (1) the development and optimization of the algorithm (*n* = 4) or (2) the validation of the algorithm (*n* = 6). This was performed using an online randomization tool (www.stattrek.com/statistics/random-number-generator.aspx (accessed on 16 June 2021)) based on earlier research [21].

### 2.3. Activity Protocols

Participants were asked to perform 14 different activities relevant and common to IC patients in and around an IC bed, including transitions between activities (Appendix A). Activities were performed in a fixed order according to four protocols:

(1) *Protocol natural*: activities were performed without further instructions in order to induce natural variation in how participants performed the activities,
(2) *Protocol healthcare provider*: activities were performed with the assistance of a healthcare provider in order to reflect the (passive) status of an IC patient, especially during the transition from one activity to another,
(3) *Protocol strict*: activities were performed strictly based on instructions on how to do so and with additional instructions if they were not performed correctly, and
(4) *Protocol bed cycling*: active bed-biking on a bed-cycle ergometer (MOTO-MED) at a self-selected speed.

Protocols 1–3 took approximately eight minutes, and Protocol 4 about four minutes.

### 2.4. Interrater Reliability of the Video Observations

Video recordings were made with a video camera of all of the activities performed by the participants (Sony, full HD (1080p), type HDR-PJ410, 50 fps). These were used for the development and optimization of the algorithm and as a reference standard for the validation of the algorithm. The camera was placed on a tripod 1.10 m from the end of the IC bed at a height of 1.50 m. For the walking activity, the camera was rotated to ensure that the participants remained in focus. During the last protocol, i.e., the bed-cycling protocol, the camera with the tripod was repositioned to the corner of the ICU room.

Two different raters (YD and ASN) separately and independently determined the type and duration of activities. The latter was calculated as the start time minus the end time of an activity from the video recordings within a 0.004 s window. For this, a video analysis and modeling tool that allowed a detailed analysis of video elements (Tracker, version 5.1.5) was used. To enhance the analysis, a distinction between ‘static’ and ‘dynamic’ activities was made. For a static activity, such as lying or sitting, the moment when the participant was completely still in the required position was selected as start time while the moment when the participant moved again was noted as the end time. For dynamic activities, such as transitions and walking, the time when the participant initiated the movement as indicated by a high velocity of postural change leading to a new activity was chosen as the start time; the moment when the participant was in a new activity was noted as the end time. Hesitations and minor repositioning of arms, legs, and clothes before or after the transition were thus disregarded. Slight limb movements during a static activity were scored as noise, whereas extensive limb movements combined with body movement were categorized as transitions as long as they occurred between different activities. If body movement did not correspond to a specific activity in the protocol, it was categorized by the raters as ‘undefinable’. 

### 2.5. Accelerometer Locations

**Figure 1:**
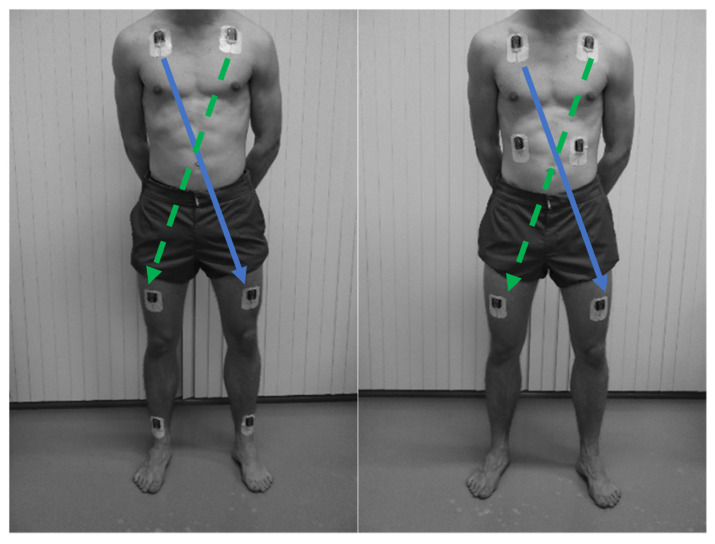
Pictures showing the accelerometer locations, with the blue arrows indicating the midclavicular line right-thigh left (MCR-TL) and green (dotted) arrows indicating the midclavicular line left-thigh right (MCL-TR) configuration used in the present study.

Six tri-axial accelerometers (ActivPAL4, ActivPALTM micro, PAL Technologies, Glasgow, UK), size, 23.5 mm × 43 mm × 5 mm, weight 9 grams, and a sampling frequency of 20–40 Hz (for this study 20 Hz was chosen) were fixed on the body with Tegaderm (Figure 1). In the present study, data from two sets of two accelerometers were used, i.e., midclavicular line left to right thigh (MCL-TR) and midclavicular line right to left thigh (MCR-TL) (Figure 1). Data forming the third set of accelerometers (i.e., rib cage or ankles) (Figure 1) will be analyzed later for different configurations in a separate study. Locations of the accelerometers used in this study were selected based on Rauen et al., 2018 [16] and Hartley et al., 2018 [15], who demonstrated that combinations of two accelerometers at these locations enabled them to distinguish between sitting, lying, and lying on the left or right side. In the current study, in contrast to Rauen et al., 2018 [16], the thigh accelerometer was placed on the anterior side of the thigh in order to avoid pressure on the skin or tissue when someone is lying on his/her side.

### 2.6. Processing of Accelerometer Data

Raw accelerometer data consisted of an acceleration signal in each axis (x, y, z). From these raw data, three variables were derived, i.e., horizontal and vertical body angles as well as a signal magnitude area (SMA). Accelerometer angles were calculated in three steps. First, the raw acceleration signals for each axis were smoothed using a three-point median filter. Second, the gravitation component (GA) representing the tilt of the accelerometer with respect to the field of gravity was extracted by applying a low pass infinite impulse response filter (IIR) with a cutoff to the acceleration signal at 0.25 Hz. Third, the tilt angles were computed as described by Fisher [22]. In order to account for sensor drift of the accelerometers, baseline corrections were made for all body angles by subtracting the offset of those that were obtained during this supine rest position. This way, horizontal tilt angles (θ, ψ) were set to zero and the vertical tilt angle (φ) to 90 in supine rest position (Appendix B) to make the body angles easier to interpret. The SMA represents the magnitude of bodily movements with a larger SMA during larger movements. To calculate the SMA, the GA was first subtracted from the raw acceleration signal from each axis which resulted in values that represent body accelerations. Hereafter, the integrals of the body accelerations were calculated for each axis using a sliding window method of one second and then summed which provided the SMA value for each window [16].

### 2.7. Development and Optimization of the Algorithm

To develop the algorithm classifying the performed IC activities at baseline, a flowchart with body segment angles and SMA values was made based on the algorithm used by Lugade et al. [13] and previous pilot measurements of our group (non-published data). Initial accelerometer body segment angles and a rest-activity-threshold were selected based on these pilot measurements. Initial accelerometer body segment angles were used to classify activities based on different body angles when the participant was performing the different activities with regard to the accelerometer locations. The rest-activity-threshold was used to classify static and dynamic activities using the SMA values and visual inspection of the SMA plots. Accelerometer and video data were aligned in time by selecting the first transition in the protocol; this was indicated by a strong increase in the SMA value as shown in the SMA plots. Data from the four participants of the development group were used to optimize body angles and the rest-activity threshold of the algorithm. After careful consideration, the activity categories ‘sitting on the edge of a bed’ and ‘sitting in a chair’ were combined into one category as these differed minimally in angles during performance. Furthermore, transitions during all of the protocols were combined into one category, i.e., ‘transitions’. In order to match the frequency of the accelerometers and the video, data from both the accelerometers and video were set to a frequency of 5 Hz. There was a visual inspection of these coded data (i.e., video data that were classified to type of activity organized per 5 Hz, 0.2 s) to check for notable differences between the two raters (YD and ASN). When these were ascertained, this was resolved by discussion and, with remaining uncertainties, by seeking the opinion of a third rater (MKN).

### 2.8. Statistical Analyses

To check on the quality of the reference standard, interrater-reliability of the video observations was determined. This was performed with the total time of activity per protocol with interclass correlations (ICCs) for all ten recorded participants (two-way mixed, absolute agreement, single measures) [23] using SPSS version 25.0. Interrater reliability was considered to be satisfactory when ICC > 0.75 [24].

Following the validation procedure of Lugade et al. [13], coded data (i.e., video data that was classified as type of activity organized per 5 Hz, 0.2 s) from one rater (rater one, YD) served as a reference, and the percentage of agreement (%Agr) was calculated as that of correctly coded activity by rater two (ASN) compared to the coded activity of rater one. In addition, the %Agr between raters on the video observations was used as a target score for developing the algorithm. For example, if the %Agr between rater one and two on a specific activity was 98%, this was subsequently also the target for the algorithm for that specific activity. The validity of the developed algorithm was determined by the %Agr on data from the validation group (*n* = 6) compared to all observations of rater one (YD). As the %Agr of the algorithm related to the video observations was not expected to be higher than the target score, the algorithm was considered to be valid if 80%Agr was reached [25].

## 3. Results

### 3.1. Participants and Protocols

Ten participants (five male, five female, mean age 45.9 years, SD 13.2), height 168–194 cm (mean 179.6 cm, SD 8.1), weight 62–94 kg (mean 76.2 kg, SD 11.7) completed all four protocols of activities. For one of them, the activities ‘sitting in a chair,’ ‘standing,’ and ‘walking’ were excluded from the natural protocol because the midclavicular accelerometer became unattached. In one other participant, a problem with the video camera led to the exclusion of ‘bed cycling with the bed head at 30 degrees’ in the bed cycling protocol. None of the participants experienced discomfort or side effects from wearing the accelerometers.

### 3.2. Interrater Reliability of the Video Observations

Interrater reliability of video observations between the two raters was high to excellent with all ICCs > 0.76 except for ‘walking’ in the strict protocol for which the ICC was 0.298 (Table 1). The %Agr of the coded activities between the two raters was very high with most above the 98% (Table 1). The lowest %Agr was determined for ‘walking’ and ‘transitions’ (86–94%) (Table 1). The percentage agreement of the undefinable activity between rater one and two was 91, 88, 78, and 97% for the natural, healthcare provider, strict, and bed-cycling protocol, respectively.

### 3.3. Development and Optimization of the Algorithm

With the algorithm run on the data of the development group, the %Agr between the algorithm and reference standard nearly reached the target scores for some static activities such as ‘supine and prone lying’ and ‘sitting’ in the natural and strict protocol (96–99%) for both accelerometer configurations (Table 1). However, activities with small differences in body angles such as ’semi lying on the right’ and ’side lying right’ did not reach the predetermined target score, as these categories were interchanged by the algorithm (Table 1). As the distinction between these two activities is of minimal clinical importance, the categories were merged into one category, i.e., ‘side-semi lying’, prior to algorithm validation on the validation group. Within dynamic activities, the %Agr between the algorithm and the video for activities such as ‘walking’ almost reached target score (84–85%) (Table 1); however, ’transition’ in the healthcare provider protocol scored very low (18–23%) for both configurations compared to the target score (93%) (Table 1).

**Figure 2:**
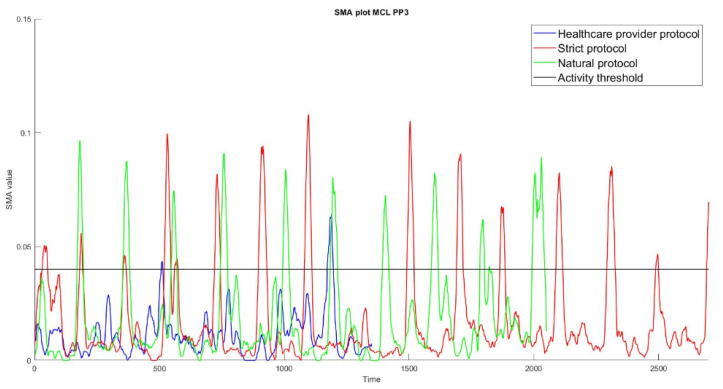
Signal magnitude area (SMA) of one participant during the performance of activities of three protocols (natural, green; healthcare provider, blue; and strict, red). The black line is used to determine the cut-off point to identify dynamic activities during the development of the algorithm. Note that the SMA value in the healthcare provider protocol (blue) is much lower compared to the other protocols.

**Figure 3:**
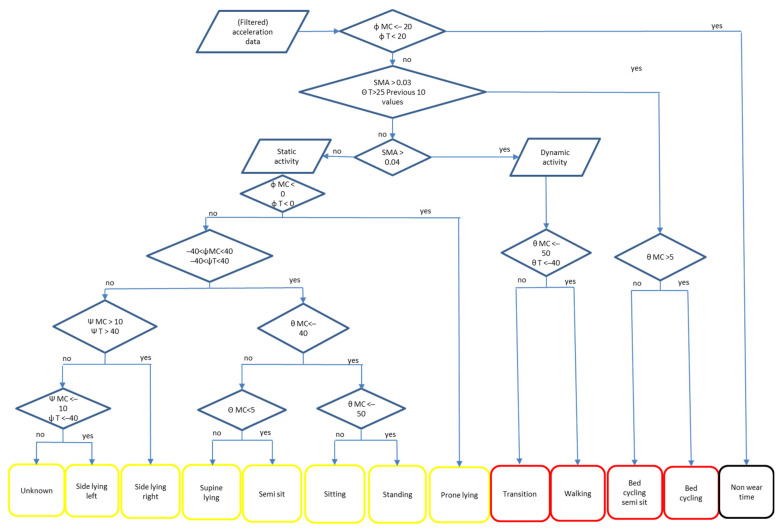
Flow chart of the developed algorithm for classification of the different activities based on body-segment-angles and SMA values of the different accelerometers. Activities in yellow and red are related to smaller and larger bodily movements, respectively. Abbreviations: SMA = signal magnitude area, MC = midclavicular accelerometer, T = thigh accelerometer, θ = theta, ψ = psi, φ = phi.

As the SMA represents bodily movements with a larger SMA during larger movements, visual inspection of the SMA plots showed that the SMA values in the healthcare provider protocol were much lower than in the other protocols (Figure 2). When the rest-activity-threshold was lowered, the %Agr for ‘transitions’ in the healthcare provider protocol increased but also resulted in an overestimation of dynamic activity in the other protocols. As ‘transitions’ in all protocols should be accurately coded by the algorithm, we decided to establish the cut-off point for the rest-activity threshold at >0.03 (Figure 2). The main goal of the SMA plots was to obtain an indication of the rest-activity-threshold required to properly classify ‘transitions’ in all protocols. For this reason, the bed-cycling protocol was not included in Figure 2 due to its more consistent SMA values. After optimizing the algorithm, the body angles and SMA values were displayed in the final flowchart (Figure 3).

### 3.4. Validity of the Developed Algorithm

**Figure 4:**
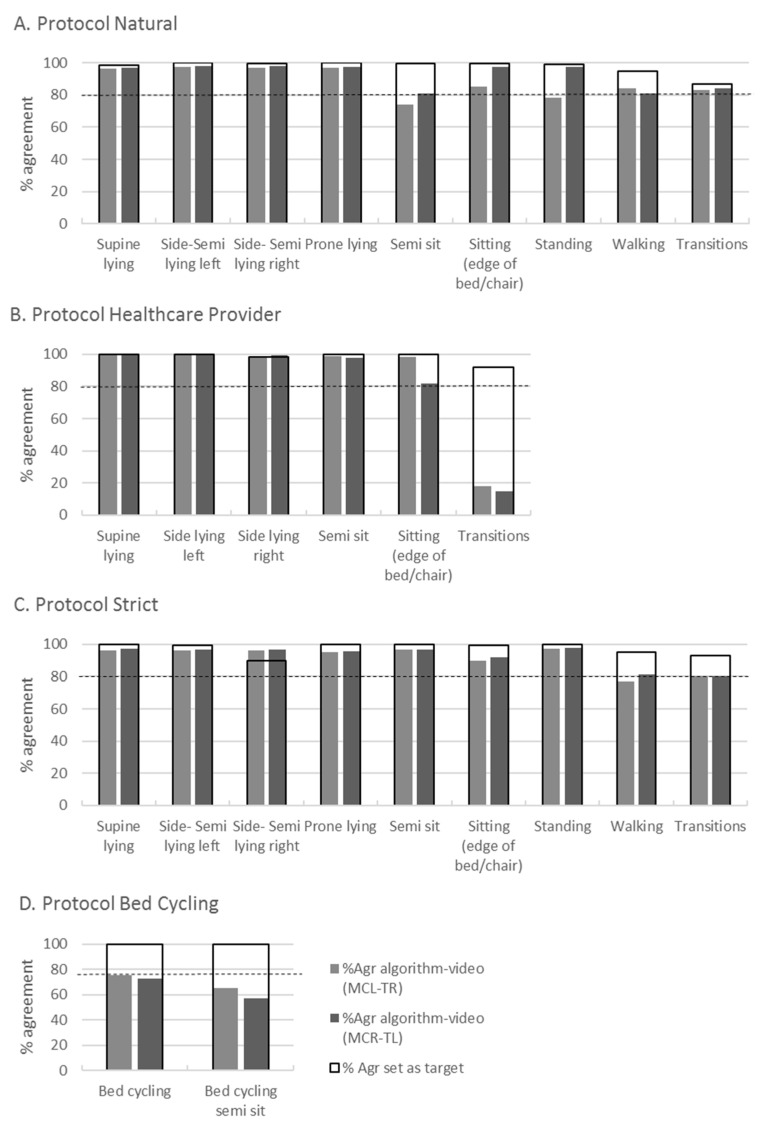
Validation of the developed algorithm showing percentage agreement of correct coded activity by the algorithm on the validation group (*n* = 6) compared to target scores based on the percentage of agreement between both video raters for all four protocols. The dotted line shows the chosen validity value of 80%. Abbreviations: %Agr, percentage agreement; MCL, midclavicular line left, MCR, midclavicular line right, TL, thigh left, TR, thigh right.

To validate the algorithm, the final algorithm (Figure 3) was run on all the data of both accelerometer configurations of the validation group (*n* = 6). The highest %Agr between algorithm and video was found for static activities such as ’prone-supine lying’, ’side lying left and right’; all were above 95% (Figure 4). For the activities ’sitting on edge of bed or chair’ and ’semi sitting’ it was 74–100% (Figure 4). The %Agr on ’transitions’ in the healthcare provider protocol was low (14–17%) while it was high on ’transitions’ in the natural and strict protocol (>80%) (Figure 4). For ‘bed cycling’, the %Agr was 57–76%. The two accelerometer configurations MCR-TL and MCL-TR had similar results (difference between 0–8%Agr) on almost all activities in the four protocols (Figure 4). Those that scored slightly lower were ‘standing’ (19%) and ‘sitting’ (12%) in the natural and (16%) healthcare provider protocol, which is not in favor of a specific configuration (Figure 4). For the absolute values of %Agr per activity per protocol for the two different configurations, see Appendix C.

## 4. Discussion

The objective of the present study was to develop and validate a dual-accelerometer-based algorithm to identify activities that are common for IC patients. The results showed that the developed algorithm based on data from two accelerometers, i.e., one accelerometer placed on chest and the other one the opposite thigh, are able to detect almost all static activities that are common and relevant for IC patients with very high agreement scores relative to the video data that served as a reference. The algorithm was also valid for identifying dynamic activities such as transitions. The only activity that had low agreement scores was ‘transitions’ with the aid of an healthcare provider. The different configurations (i.e., left and right diagonal) of the accelerometers showed similar results, implying that both diagonals can be used.

Just as in other studies using two accelerometers [12,13,16,26], we were able to correctly identify different activities, such as lying and sitting. This is essential, as IC patients spent most of their time engaged in these activities [11]. Our study adds to others in that our developed algorithm can identify additional activities, such as transitions from lying on the left to right side and bed cycling. An assessment of activities is important as, until now, detailed information on the performed physical activity of IC patients during an ICU stay has been lacking. Furthermore, although IC patients are not highly active, a recent study found significant variations in physiological responses to different types of activities [2] indicating that an assessment of all types is immensely important. To enhance rehabilitation in IC patients, an appropriate amount of physical activity is essential [27], and objective measurements about the type, intensity, frequency, and duration of performed activity by IC patients are essential [6]. Therefore, with this detailed information provided by our algorithm, the amount of physical activity that is required to optimize rehabilitation can be studied more thoroughly.

In this study, we chose to manually develop and optimize the algorithm based on body angles and SMA values using the raw data from two tri-axial accelerometers. Recently, many different classifiers have become available, such as machine learning algorithms, that appear to be promising for correctly identifying physical activity [17,28,29]. However, the usefulness of applying them in a clinical setting requires some awareness, as the optimization process is a kind of black box and cut-off points for identifying physical activity may vary depending on the conditions [28,29]. Moreover, as described in detail in a recent study [17], there are many different ways to estimate and validate algorithms; however, a comparison of results from studies is difficult because not all algorithms are described transparently. Using a machine-learning algorithm to identify physical activity is a data-driven method. Stated differently, it can develop an optimal model based on the given (sometimes very large) dataset and is effective to use on a large scale, but, just as in other models, it can only be as good as the quality of the underlying data. Nevertheless, a manually developed algorithm has some advantages, because it is both data- and logic-based, which makes the mechanism of the algorithm and the results easier to understand. This is important because adjustments to it must be clinically relevant in order to improve it so that it captures body movement relevant to a particular population. Consequently, we chose to optimize the algorithm based on video data with a very short window, to ensure that body angles fit the performed activity and not only to ensure increasing %Agr. Therefore, during the development, activities such as sitting and sitting on the edge of the bed were included separately. However, since these categories were interchanged by the algorithm, as the person is sitting and body angles were somewhat the same in both cases, they were merged into one category.

We used the SMA value to distinguish between static and dynamic activities, as this has been shown to have good sensitivity and specificity for distinguishing between these types of activities [30]. However, identifying dynamic activities such as ‘transitions’ in the healthcare provider protocol was more difficult and resulted in lower %Agr scores. A further analysis of the data in the healthcare provider protocol showed that the absolute SMA values of ‘transitions’ in it were relatively low compared to those in the natural and strict protocol (Figure 2). Selecting a lower rest-activity threshold (lower SMA value) resulted in a higher %Agr for ‘transitions’ in the healthcare provider protocol; however, such a lower threshold would have also resulted in an overestimation of the total amount of dynamic activity in the other protocols. Therefore, an SMA value of 0.04 was chosen while, in other studies in other settings, SMA values of 0.135 [13], 0.2 [16], or even 0.80 [13] were used. These findings indicate that selecting the appropriate SMA value to identify clinically relevant activities depends heavily on the situation and population for which the algorithm is being created.

In addition, regarding the lower %Agr score for ‘transitions’ in the healthcare protocol, results showed that their duration was very brief (1–2 s) while transitions such as transfer to chair performed by the participant itself in the other protocols took approximately 3–5 s. Short activity bouts were difficult to identify as higher agreement can be achieved with longer activity tasks [13].

We found all ICCs between video-observers were sufficient except for one, i.e., ‘walking’ in the strict protocol. In-depth analyses indicated that it was due to one single video in which the two observers significantly deviated (2 s) from the amount of time that the activity ended and the transition to sit in the chair began. Without this one observation, the ICC for walking in the strict protocol was 0.67 and would have been sufficient. Furthermore, our time frame for classification was 0.004 s, while, in other studies, this was much longer—for example, one second [13] and one minute [14]. As a consequence, categorizing activities was most difficult at the beginning and end of an activity. Nevertheless, we are able to detect short activities, such as turning in bed.

In this study, healthy individuals performed the ICU-related activities of the four protocols. Theoretically, it would have been optimal if the validity of the new method had been developed based on observations in IC patients. In practice, however, this would mean that ICU patients’ physical activities would have to be monitored every minute by an observer with an activity diary and/or with video. Both ethically and organizationally, this was not an option. Therefore, the choice was made to use healthy individuals and mimic the setting of ICU patients as much as possible. The various activities of the protocols were chosen based on the literature and on input from highly experienced ICU staff. The latter input in particular was important, and led to the inclusion of activities performed with and without instructions, and even more importantly, with the help of healthcare providers, which has not happened in previous similar studies [13,16,17,26]. It is possible that the validity of the new method is slightly lower when the activities have been performed by IC patients. Reasons for this include that some activities could be misclassified by the algorithm, for example during nursing activities such as washing. This could lead occasionally to the over- or underestimation of a specific activity, for instance more time being spent in dynamic than in static activities. However, we do not think this is a problem, as our algorithm was found to be valid even with many very high agreement scores.

Another issue is that the activities chosen may be performed differently by IC patients than by healthy individuals, such as when a patient is slumped into a chair due to limited trunk balance or walks with a walking aid. In that case, the SMA values and combination of angles may not fall within the predefined ranges for these activities. The algorithm will then classify more time as ‘unknown’. This does not influence validity as such, but it does have implications for identifying real-life activities performed by IC patients. To cover all activities in practice, an additional step in the development of the algorithm might be needed. Currently two studies are being conducted, one in a burn ICU and one in a general ICU using the algorithm in combination with an activity diary. Results from these studies will give us insight into which activities are not identified.

## 5. Strengths and Limitations

A strength of this study is that we chose to place the thigh accelerometer at the front of the thigh rather than on the lateral side as was performed by Rauen et al., 2018 [16]. This location is more useful in bedridden patients because it avoids pressure when lying on their side. Second, the algorithm developed in this study is appropriate for both tested accelerometer configurations (both diagonals) to identify different activities. This finding implies that, if an IC patient has lines, devices, or wounds at one of the accelerometer locations, a shift of locations can be made without the outcome being different. Third, all participants performed all four different protocols, including one for which transitions were performed with the aid of a healthcare provider. Consequently, all of the different activities were performed multiple times, and variation in how the separate activities were performed by each individual is included which improves external validity for use in an IC setting. Fourth, the activities in the different protocols were widespread (from lying down to walking) which allows this algorithm to also be applied when patients become more active. In this context, this algorithm can provide insight into activities performed throughout the course of hospitalization. Fifth, we randomized the participants’ data into a development and a validation group. This afforded us the opportunity to monitor whether the algorithm that was developed would also be valid with an independent set of samples and to ensure that this algorithm can be applied in the ICU setting. Sixth, activities were classified based on video recordings observed by two different raters. Very high ICC values were achieved, guaranteeing the reliability of activity classification.

There are, of course, also limitations to this study. First of all, we did not instruct participants to walk or bed cycle at a certain speed. As a result, it was more difficult to develop the algorithm, and it might have missed part of these activities if these fell beyond the cut-off points. Nevertheless, our algorithm was able to identify these activities even though participants chose their own speed and varied while performing the activities. Another limitation is that the accelerometers and video data did not begin at the exact same time, as the accelerometers had to be installed beforehand. Therefore, the first peak in the SMA value was used to align the accelerometer data and the video observations. Thus, the first transition of the protocol was used. However, especially in the healthcare provider protocol, the transitions were very short (only 1–2 s) which made aligning the data challenging. Though this is a technical limitation, it can only have a positive effect on the validity of the algorithm in a genuine setting as the agreement scores, and thus the validity, might be higher than currently indicated with perfect time alignment. Finally, the activities were performed by healthy participants. When IC patients perform the activities that are incorporated in the algorithm, they can be identified and quantified in IC patients.

## 6. Conclusions

The dual-accelerometer-based algorithm developed in this study to identify and quantify activities that are common for IC patients, with video as reference, was found to be valid. It identifies prone lying, supine lying, lying on the left or right side, sitting, semi-sitting, standing, walking and bed cycling. Both examined accelerometer configurations on the chest and opposite leg showed similar results, which implies that both diagonals can be used and interchanged if clinically necessary. In future clinical studies, this dual-accelerometer-based algorithm can be utilized to provide objective measurements for IC patients over periods of many days regarding the type, frequency, and duration of the different activities that are performed.

## Figures and Tables

**Table 1:** Interrater reliability of video analysis, percentage agreement between two raters on the coded activities, and percentage agreement of the algorithm related to the video on the development group data.

	Duration of Activities in Seconds with ICC and CI Between Two Raters (Mean and SD)				%Agr between Two Raters on Coded Video Data	%Agr between Algorithm and Video	
Protocol Natural	Rater 1 (s)	Rater 2 (s)	ICC	95% CI	%Agr *	%Agr MCL-TR (*n* = 4)	%Agr MCR-TL (*n* = 4)
Supine lying	37.12 (7.74)	36.74 (7.60)	0.98	0.93–0.99	98.5	99.1	96.7
Side lying left	31.4 (2.35)	31.92 (2.40)	0.92	0.68–0.98	99.8	72.8	73.4
Side lying right	31.62 (1.83)	32.02 (2.07)	0.96	0.69–0.99	99.8	73.6	98.3
Prone lying	32.76 (1.96)	33.08 (2.02)	0.97	0.80–0.99	100	98.0	98.2
Semi sit	33.52 (1.94)	33.98 (2.11)	0.95	0.62–0.99	99.5	80.1	70.1
Semi lying left	31.62 (4.02)	32.04 (4.12)	0.97	0.90–0.99	99.7	99.0	74.4
Semi lying right	33.30 (2.38)	33.72 (1.90)	0.94	0.74–0.98	99.6	74.7	50.3
Sitting (edge of bed or chair)	32.98 (1.72)	33.37 (1.48)	0.86	0.65–0.95	99.6	99.1	98.3
Standing	36.11 (5.69)	36.44 (5.90)	0.99	0.95–0.99	99.0	98.3	99.4
Walking	6.50 (0.79)	6.46 (1.17)	0.76	0.27–0.94	94.6	85.4	89.9
Transitions	3.39 (1.85)	3.35 (1.97)	0.87	0.81–0.95	86.9	85.1	86.9
**Protocol Strict**							
Supine lying	33.06 (2.59)	33.14 (2.56)	0.99	0.99–0.99	99.8	98.3	97.7
Side lying left	31.06 (1.43)	31.34 (1.45)	0.96	0.73–0.99	99.8	99.2	98.2
Side lying right	31.82 (1.87)	32.26 (1.72)	0.88	0.59–0.97	99.5	72.4	96.7
Prone lying	32.72 (1.74)	33.18 (1.63)	0.91	0.56–0.98	99.9	99.1	96.4
Semi sit	32.76 (2.79)	33.50 (3.32)	0.94	0.62–0.99	99.8	98.7	73.1
Semi lying left	33.04 (2.38)	33.52 (1.81)	0.89	0.62–0.97	99.1	96.2	94.6
Semi lying right	33.28 (4.90)	33.56 (5.01)	0.99	0.96–0.99	99.9	74.6	74.5
Sitting (edge of bed or chair)	33.24 (2.58)	34.02 (2.37)	0.80	0.51–0.92	99.4	99.4	97.9
Standing	34.54 (4.55)	35.36 (4.25)	0.97	0.71–0.99	99.9	98.4	98.9
Walking	6.16 (0.83)	6.36 (0.98)	0.30	−0.41–0.77	95.1	83.6	88.9
Transitions	2.95 (1.38)	3.26 (1.43)	0.81	0.72–0.87	92.8	84.5	82.9
**Protocol Healthcare Provider**							
Supine lying	38.90 (5.13)	39.02 (5.00)	0.99	0.98–0.99	99.7	88.0	100.0
Side lying left	32.82 (2.13)	33.04 (2.09)	0.98	0.91–0.99	99.7	99.7	88.0
Side lying right	33.64 (2.31)	33.32 (2.86)	0.80	0.40–0.95	98.3	96.4	98.7
Prone lying	-	-	-	-	-	-	-
Semi sit	37.42 (3.89)	37.84 (3.88)	0.98	0.91–0.99	99.8	100	99.9
Semi lying left	-	-	-	-	-	-	-
Semi lying right	-	-	-	-	-	-	-
Sitting (edge of bed or chair)	33.85 (2.80)	34.35 (3.02)	0.97	0.80–0.99	99.8	97.9	88.5
Standing	-	-	-	-	-	-	-
Walking	-	-	-	-	-	-	-
Transitions	1.85 (1.36)	2.37 (1.52)	0.79	0.45–0.91	92.0	23.9	18.5
**Protocol Bed Cycling**							
Bed cycling	130.36 (3.55)	130.46 (3.94)	0.98	0.94–0.99	99.6	61.1	91.1
Bed cycling semi sit	135.44 (13.36)	137.05 (13.55)	0.96	0.86–0.99	99.9	65.1	48.9

Abbreviations: %Agr, percentage agreement, MCL: midclavicular line Left, MCR: midclavicular line right, TL: thigh left, TR: thigh right; * percentage was also used as target for developing the algorithm on the data of the development group (*n* = 4).

## Data Availability

Essential data relevant to the findings of this study can be found in the Appendix. Additional data supporting the findings of this work are available upon reasonable request by contacting the corresponding author.

## Abbreviations

IC	Intensive Care
ICC	Intra Class Correlation Coefficient
MCL-TR	midclavicular line left to right thigh
MCR-TL	midclavicular line right to left thigh
SMA	Signal Magnitude Area
%Agr	Percentage Agreement
MCL	Midclavicular line Left
MCR	Midclavicular line Right
TL	Thigh Left
TR	Thigh Right
CI	Confidence Interval
GA	gravitation component

## Appendix A

**Table A1:** Description and Duration of the Different Activities and Transitions for the Four Different Protocols.

Protocol Naturally	t (s)	Protocol Strict	t (s)
lying in bed (supine)	30	lying in bed (supine)	30
* transition supine-side lying left*		* transition supine-side lying left*	
side lying left	30	side lying left	30
* Transition side lying left-side lying right*		* transition side lying left-supine*	
side lying right	30	lying in bed (supine)	30
* transition side lying right-prone lying*		* transition supine-side lying right*	
prone lying	30	side lying right	30
* transition prone lying-semi sit*		* transition side lying right-supine*	
semi sit	30	lying in bed (supine)	30
* transition semi sit-semi lying left side*		* transition supine-prone lying*	
semi lying left side	30	prone lying	30
* transition semi lying left-semi lying right*		* transition prone lying-supine*	
semi lying right side	30	lying in bed (supine)	30
* transition semi lying right- edge of bed*		* transition supine-semi sit*	
edge of bed	30	semi sit	30
* transition edge of bed-sitting in a chair*		* transition semi sit-semi lying left side*	
sitting in a chair	30	semi lying left side	30
* transition sitting in a chair-standing*		* transition semi lying left-semi lying right*	
standing	30	semi lying right side	30
* transition standing-walking*		* transition semi lying right- supine*	
walking for 2 m, turn and return		lying in bed (supine)	30
		* transition supine-edge of bed*	
		edge of bed	30
**protocol healthcare**	**t (s)**	* transition edge of bed-sitting in a chair*	
lying in bed (supine)	30	sitting in a chair	30
* transition supine-side lying left*		* transition sitting in a chair-standing*	
side lying left	30	standing	30
* transition side lying left-side lying right*		* transition standing-walking*	
side lying right	30	walking for 2 m, turn and return	
* transition side lying right-semi sit*			
semi sit	30		
* transition semi sit-edge of bed*		**protocol bed cycling**	**t (s)**
edge of bed	30	bed cycling with the head of bed on 10 degrees	120
* transition edge of bed-sitting in a chair*		* head of bed till 30 degrees*	
sitting in a chair	30	bed cycling with the head of bed on 30 degrees	120

Abbreviations: t time, s seconds.

## Appendix B

**Table A2:** Classification of Activities Based on Body-Segment-Angles and SMA Values from Two Three-Axis Accelerometers (Placed on Thorax and Opposite Leg).

Different Activities	Assumptions	
Supine lying	T phi > 0 B phi < 0 T psi < 40 T psi > −40 B psi < 40 B psi > −40 T theta > −40 T theta > 5	
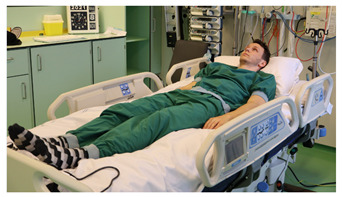
2. Side lying left	T phi > 0 B phi < 0 T psi > 40 T psi < −40 B psi > 40 B psi < −40 T theta > −15 T theta < −40 T psi < −15 B psi < −50	
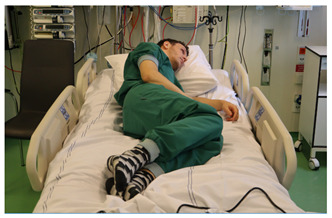
3. Side lying right	T phi > 0 B phi < 0 T psi > 40 T psi < −40 B psi > 40 B psi < −40 T theta > −15 T theta < −40 T psi > 10 B psi > 50	
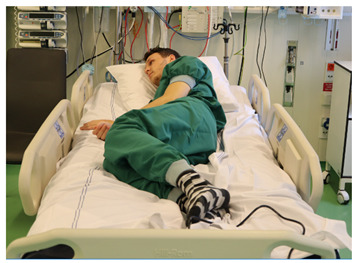
4. Prone lying	T phi < 0 B phi < 0	
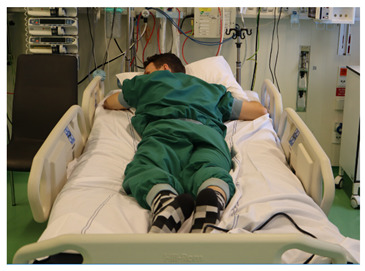
5. Semi sit	T phi > 0 B phi < 0 T psi < 40 T psi > −40 B psi < 40 B psi > −40 T theta > −40 T theta < 5 T theta > −40	
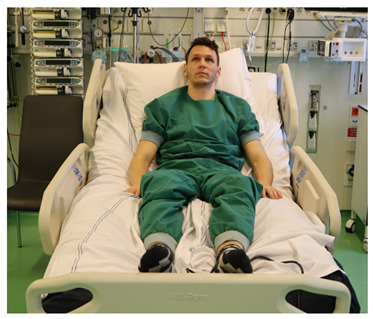
6. Semi lying left	T phi > 0 B phi < 0 T psi > 40 T psi < −40 B psi > 40 B psi < −40 T theta < −15 T theta > −40 T psi < −15 B psi < −45	
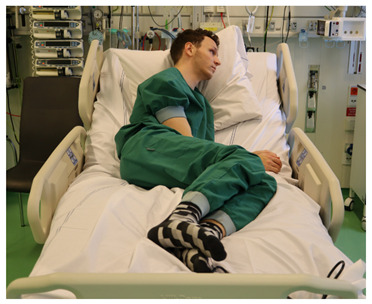
7. Semi lying right	T phi > 0 B phi < 0 T psi > 40 T psi < −40 B psi > 40 B psi < −40 T theta < −15 T theta > −40 T psi > 15 B psi > 45	
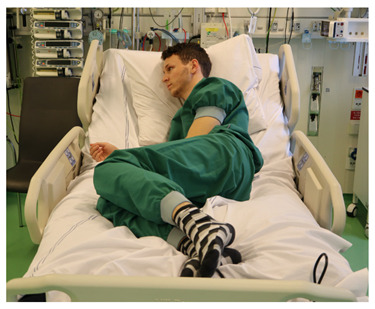
8. Sitting (edge of bed)	T phi > 0 B phi < 0 T psi < 40 T psi > −40 B psi < 40 B psi > −40 T theta <−40 B theta >−50	
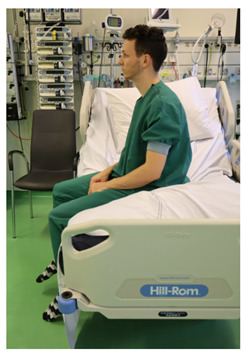
9. Sitting (chair)	-	
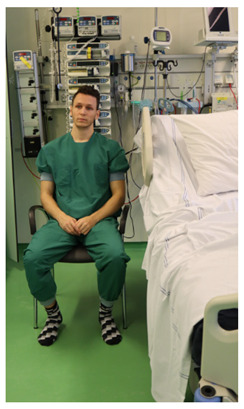
10. Standing	T phi > 0 B phi < 0 T psi < 40 T psi > −40 B psi < 40 B psi > −40 T theta <−40 B theta <−50	
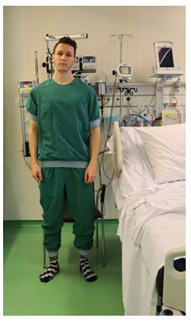
11. Walking	T SMA > 0.04 T theta <−50 B theta <−40	
12. Bed cycling (head of bed 10 degrees)	B SMA > 0.03 T theta > 5 B theta > 25 Previous 10 values either 12 or 13	
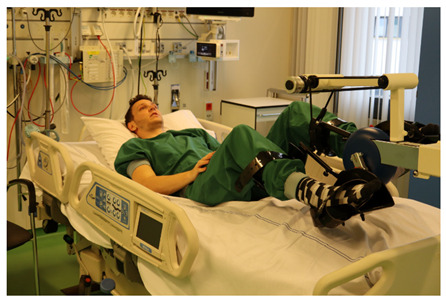
13. Bed cycling semi sit (head of bed 30 degrees)	B SMA > 0.03 T theta < 5 B theta > 25 Previous 10 values either 12 or 13	
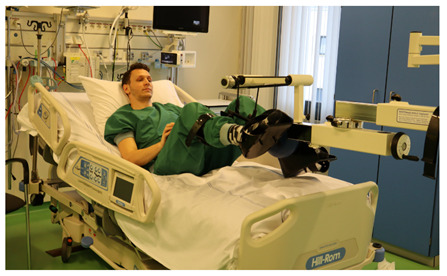
14. Transitions	T SMA > 0.04 B theta > −40	

Abbreviations: T, accelerometer placed at het thorax, B, accelerometer placed at the leg, SMA, signal magnitude area.

## Appendix C

**Table A3:** Absolute Values Represented as %Agr of the Algorithm Related to the Video for the Validation Group (*n* = 6).

Protocol Natural			
Supine lying	98.5	96.5	97.0
Side-semi lying left	100.0	97.6	98.2
Side-semi lying right	99.6	96.9	98.1
Prone lying	100.0	97.1	97.6
Semi sit	99.5	74.3	80.7
Sitting (edge of bed or chair)	99.6	85.0	97.3
Standing	99.0	78.4	97.7
Walking	94.5	83.9	81.1
Transitions	86.9	83.1	84.1
**Protocol Strict**			
Supine lying	99.8	96.4	97.1
Side-semi lying left	99.4	96.2	96.8
Side-semi lying right	89.7	96.0	96.9
Prone lying	99.9	95.3	95.9
Semi sit	99.8	96.6	96.5
Sitting (edge of bed or chair)	99.4	89.9	92.1
Standing	99.9	97.5	97.7
Walking	95.1	77.0	81.3
Transitions	92.8	80.0	80.3
**Protocol Healthcare Provider**			
Supine lying	99.7	99.5	100.0
Side lying left	99.7	99.7	99.6
Side lying right	98.3	98.7	99.6
Prone lying	-	-	-
Semi sit	99.8	99.0	97.8
Sitting (edge of bed or chair)	99.8	98.3	81.8
Standing	-	-	-
Walking	-	-	-
Transitions	92.0	17.9	14.7
**Protocol Bed Cycling**			
Bed cycling	99.6	75.7	73.0
Bed cycling semi sit	99.9	65.4	57.3
